# How much is adequate staffing for a nursing home? Forecasting daily service need of its case mix

**DOI:** 10.1007/s10729-025-09740-8

**Published:** 2026-03-24

**Authors:** Shujin Jiang, Mingyang Li, Nan Kong

**Affiliations:** 1 Edwardson School of Industrial Engineering, Purdue University, West Lafayette, IN 47907 USA; 2 Department of Industrial and Management Systems Engineering, University of South Florida, Tampa, FL 33620 USA; 3 Weldon School of Biomedical Engineering, Purdue University, West Lafayette, IN 47907 USA

**Keywords:** Healthcare analytics, Nursing home, Service need forecasting, Bayesian sequential learning

## Abstract

Staffing adequately in an economical manner is vital to nursing homes (NHs) in the United States. NHs strive for providing resident-centered differentiated service to their changing and diverse residents and service cases. In this paper, we present a novel Bayesian forecasting method to predict acuity category-specific resident volume and caregiver type-specific staff time on a daily basis for NHs. We utilize the Minimum Data Set (MDS) alongside the Resource Utilization Group (RUG) guidelines according to residents’ acuity and the Patient Driven Payment Model (PDPM) specifications on recommended staff time in response to their staffing need, to generate two time series on daily NH service need, i.e., resident volume of each acuity group and staff time of each caregiver type in the entire facility, respectively. Given that the two multi-dimensional time-series above are nonstationary with potential correlations between dimensions, we propose prediction models with time-varying latent states to capture the dynamic patterns in the data and incorporate shared information between different categories. Specifically, we introduce a generalized mixture model to predict the discrete-valued resident volume and a generalized linear model to predict the continuous-valued staff time. Further, we propose a unified Bayesian estimation framework that allows convenient sequential updating and embed it in a forecasting procedure with rolling window to better capturing the nonstationarity inherent in the data. We demonstrate the superiority of the proposed model-based Bayesian forecasting method by comparing its performance against benchmark methods, using data from representative NHs.

## Highlights

- Improve accuracy in predicting daily acuity category-specific resident volume andcaregiver type-specific staff time for nursing homes (NHs) and their changing residentmix.
- Propose a Bayesian sequential learning framework to enhance prediction accuracy inface of nonstationary time-series data.
- Develop a conversion protocol to quantify service need for NH resident mix withregularly reported, standardized NH assessment data.
- Introduce MDS 3.0 to the operations management research community so thatresearchers can help enhance the forecasting capability of NHs by utilizing this dataresource, which is critical to their business and operations management success.

## Introduction

Nursing homes (NH) in the United States (US) provide the most comprehensive set of professional care a person can receive outside hospitals. The care includes a range of coordinated medical, personal, and social services to meet the needs of residents who are chronically ill or disabled [44]. Services at a nursing home typically include nursing care, 24-hour supervision, and assistance with everyday activities. In some NHs, rehabilitation services, such as physical, occupational, and speech therapy, are also available [34].

NH residents suffer from diverse chronic diseases and functional limitations, and their service need can differ significantly. These needs are often related to their acuity (i.e., clinical complexity and ability to perform daily activities). Moreover, for some residents, the needs can vary significantly over time [46].

To NHs, it is important to incorporate residents’ diverse, changing acuity and needs when developing care plans for them. Since it is not viable from an operational standpoint to change the work schedule of NH staff on the fly, it is important for NHs to be able to anticipate changes in their mixed resident volume and service need over each decision epoch (typically bimonthly or monthly). In addition, accurate forecasting of seasonal demand would allow NHs to adjust their staffing level proactively.

Without a good forecasting tool, NH staffing in current practice is either determined by the experience of NH administrators in charge [35] or by the minimum staff-to-resident ratio requirements enforced by federal/state agencies [18]. However, several studies have argued that the current minimum requirements need to be redesigned as a one-size-fits-all strategy may not translate well to satisfactory care outcomes at diverse NHs and among residents of diverse needs [20, 22, 29]. Recent studies have further suggested that NHs must take into account resident acuity when determining the adequate staffing levels to meet the needs [23].

In this paper, we present a model-based Bayesian forecasting method that utilizes individual-level clinical assessment data to provide accurate forecasts on future NH service need. We process individual resident’s assessment data in the Minimum Data Set (MDS) to generate time-series measures on NH service need. We offer predictions on daily resident volume and staff time at a higher level of granularity. This will allow NH managers to adjust staffing with improved patient- and provider-centeredness. To achieve it, we propose the use of Bayesian forecasting techniques to deal with the complexities in the time series data. Subsequently, the anticipated improvement on the service need forecasting can lead to better staffing management, improved health outcomes and service satisfaction in residents, and elevated work morale for caregivers. Research has found that more adequate staffing can lead to lower staff turnover rates and better resident outcomes [8–10, 51].

### Background on the data

The Nursing Home Reform Act, which was passed in 1987, established federal quality standards for NHs. With the act, NHs that are certified to participate in Medicare and Medicaid are mandated by the US Centers for Medicare and Medicaid Services (CMS) to meet these standards for residents who are enrolled in Medicare or Medicaid programs. Medicare and Medicaid are the two main public funding resources for health care. Medicare is federal health insurance for anyone age 65 and older and some people under 65 with certain disabilities or conditions. Medicaid is a joint federal and state program that gives health coverage to some people with limited income and resources.

As part of the federally mandated process, NHs must conduct a resident assessment and care screening for their residents periodically to evaluate their resident needs. The data are collected by the CMS and managed with the so-called minimum data set (MDS) [34]. MDS Version 3.0 Resident Assessment and Care Screening, implemented in current practice, a powerful tool for implementing standardized assessment and facilitating care management in NHs. The assessments are conducted when a resident is first admitted to NH, at regular intervals during their stay, and upon their discharge from the facility. MDS data on resident functionality is gathered from nursing staff documentation at their work. Upon the completion of an assessment, the assessment information leads to the acuity (re)categorization, which is mainly dependent upon residents’ clinical complexity and cognitive functions. Meanwhile, the admission and discharge information helps specify the exact cohort of the residents on any given day. Thus, there is an up-to-date resident mix in each NH based on all residents’ acuity categories, which is often termed in the nursing literature resident census. We use the daily acuity category-specific resident volumes as the first observational quantity of interest.

Following the CMS guidelines [17], a resource utilization group (RUG) is further assigned to each resident to specify the level of skilled service they require. This classification system takes additional consideration on residents’ well-being, independence, and their mental health condition. It is used by NHs to determine the level of reimbursement to request from CMS on the Medicare and Medicaid beneficiaries they care for. The RUG Version IV, used in current practice, helps CMS make fair comparisons between NHs and enables better tracking of resident conditions at each NH. Nursing staff, as the core service providers at NH, provide much of direct medical treatment and personal assistance to the residents. In current practice, NHs, mandated by the CMS, must achieve a reasonable resource utilization level (e.g., sufficient number of NH beds), depending on resident’s RUG [21]. This resource utilization based classification system, maps the 6 non-rehab-focused acuity categories alongside additional information into 48 RUGs; see Fig. 8 in Appendix.

Nevertheless, the RUG-based staffing standard may still not be sufficient to meet the detailed needs of NH residents, especially those with complex care needs. CMS thus makes additional recommendation on need-based specifications on NH staffing for different types of caregivers. Typically, in a NH, three types of caregivers, i.e., Registered Nurses (RNs), Licensed Practical Nurses (LPNs), and Certified Nursing Assistants (CNAs), are staffed to provide comprehensive care service and personal assistance to the residents. RNs play an important role in residential service care (e.g., giving medicines to residents, and monitoring and recording their health conditions). CNAs are important in assisting residents with daily living activities (e.g., transferring, moving, and feeding residents). LPNs are responsible for major nursing care duties (e.g., measuring vital signals and administering IV injections of residents).

The patient-driven payment model (PDPM) [11], currently implemented, utilizes MDS 3.0 as the basis and takes the RUG-IV classification further; see Table 2 for CMS-recommended staff time in Appendix. It is designed to take into account specific needs and care goals of each NH resident, and it is intended to establish a nationwide benchmark on daily staffing required for high-quality care delivery by the three types of caregivers collectively. Following this CMS implementation, we considered daily facility-wide staff time (in hours) of each type of caregivers as the second quantity.

### Research overview

We develop an data analytics pipeline to facilitate the processing of each NH’s MDS for its patient volume and staff time forecasting. The NH resident volume forecasting helps infer NH capacity planning decisions. We process the MDS data to generate daily resident volume data for each acuity category in the NH of interest. To further support operational decisions on caregiver staffing and scheduling at the NH, we differentiate individual residents’ service need based on their acuity groups in forecasting adequate staff time. We follow the RUG guidelines, and then apply the PDPM to synthesize CMS-recommended daily staff time for each caregiver type, which is based on the daily volumes among the RUGs in the NH.

For the forecasting, we develop a novel Bayesian forecasting method that models the temporal trends of both quantities on NH service need (resident volume and staff time), which addresses various data complexities. Through preliminary investigation, we identify several key features inherent in the time-series data, which presents challenges to forecasting. First, due to the highly stochastic nature of NH resident admissions with varied acuity levels over time, both the discrete and continuous data exhibit non-stationary patterns over time. Using time-invariant modeling parameters in conventional time-series modeling becomes inadequate. We thus propose Bayesian latent variable models with time-varying latent states to dynamically capture the non-stationary evolving patterns of the resident service need. Further, a unified Bayesian estimation and sequential updating algorithm are further proposed to enable convenient sequential model updating when new discrete/continuous data becomes sequentially available. Second, in the census data, many RUGs may often have excessive zeros, making the conventional discrete modeling assumption inappropriate. We introduce a mixture modeling technique to capture the excessive zeros in the developed discrete census model. Finally, due to the potential correlation between resident groups or caregiver types, we further borrow information from other groups/types to enhance the group/type-specific service need forecasting.

To assess the performance of our proposed forecasting method, we use individual-level assessment data of Medicaid-funded residents in a Midwest US state from the state’s MDS 3.0 database. We compare their performance against that of state-of-the-art demand forecasting methods, with and without considering the data nonstationarity. We carry out these comparative evaluations using the back-testing method. We obtain insights through the numerical experiments on method selection and provide guidance on how to use our method in different data cases.

Our contributions are listed as follows:

- We enhance the capability of nonstationary time series forecasting by leveraging a generalized prediction modeling framework and proposing a Bayesian sequential learning algorithm for updating model parameters over time. In addition, we present an impactful use case to the research with de-identified patient data and sequential updating codes made available.
- We present to the Operations Management research community a well-packaged stochastic service demand generator. This can be useful when investigating the application of rolling-horizon optimization approaches to shift-based staffing and scheduling. For example, many service organizations are interested in proactively adjusting their hiring decisions/policies for organizational efficiency while maintaining satisfactory service.
- We propose a data analysis pipeline to aggregate individual assessment data in MDS into daily resident-mix service need estimates. This can be used to help benchmark NH need assessment data processing and analysis, which will improve NH resource management. Meanwhile, this pipeline automates the RUG grouping process, which can augment NH care outcomes research.

The remainder of this paper is organized as follows. In Section 2, we review the relevant literature. In Section 3, we present the technical details on MDS data processing. In Section 4, we present the prediction models and learning algorithms together with benchmark forecasting methods. In Section 5, we generate case studies based on various NHs. We report the results of comparing different forecasting models and methods and discuss our insights into the association between the data and method selection. Finally, we draw conclusions and outline future research in Section 6.

## Related work

In this section, we review the literature in three relevant aspects: (1) how MDS data has been used; (2) demand forecasting models and methods in healthcare OM; and (3) Bayesian forecasting for NH service need.

### Use of MDS

MDS has become a valuable resource for research studies with the aim of improving care quality, resource allocation, and quality measurement in NHs. Hawes et al. [24] emphasized that MDS has the potential to significantly impact long-term care policy of its emphasis on clinical utility. Arling et al. [4] analyzed the variation in direct care resource use among residents based on the RUG classification system. The study suggested new criteria and quality indicators be developed to better reflect the relative costs of caring for the residents. Shin and Scherer [45] and Toth et al. [52] assessed the methodological validity of using MDS data to characterize perineal dermatitis risk factors by comparing the MDS data with data from NH chart records.

Moreover, MDS data has been utilized in various studies to model NH residents’ health condition and assess the quality of their service; see e.g., [39, 40, 57]. Our work extends the use of MDS data by incorporating the RUG classification system and a patient-driven payment model, which bridges healthcare services research with operations management to quantify both need-based resident volume and staff time for arbitrary resident mix.

### Demand forecasting in healthcare OM

The problem of demand forecasting has been studied extensively in the healthcare operations management literature, e.g., bed occupancy and admission count in the context of emergency medicine [3, 25, 27, 48]. For this problem, the use of univariate methods under normal and stationary assumptions is a widely recognized approach at present [1, 37]. In addition, Schweigler et al. [43] utilized the exponential smoothing approach to forecast emergency department crowding. Claudio et al. [14] applied a modified clustering method to enhance the prediction of outpatient cancer clinic demand. Perry et al. [38] employed exponentially weighted moving averages to improve the accuracy in forecasting emergency department visits for respiratory illness. Moreover, there are studies that adopt multivariate forecasting models. Jones et al. [26] and Kam et al. [27] reported that the use of a multivariate model could provide a more accurate forecast of emergency inpatient flow, compared to the standard univariate models.

Nevertheless, numerous models that are applied to continuous time series data are inadequate to model discrete-valued data (e.g., the need-based case-mix resident volume in our study). In addition, the census data contains many zeros, making it even more challenging to model accurately using a continuous distribution. Davis [15] presented a collection of modeling techniques for discrete-valued time series data, such as binary and categorically valued.

Furthermore, in the above healthcare OM studies, normal and stationary assumptions are often made on the statistical properties of the data. These methods work fine for stationary time series data. However, in our study, the NH data presents nonstationarity. Thus, the above methods may not produce accurate forecasts, which motivates us to explore the use of Bayesian forecasting.

### Bayesian forecasting

To address the issue of nonstationarity inherent in our data, we adopt Bayesian forecasting, which is an approach that treats unknown parameters as random variables and further utilizes subjective probability distributions to quantify the uncertainties of the random variables given a finite sample size of data available [41]. This is in contrast to the frequentist approach to inference. To estimate unknown variables related to system states of interest, we employ a Bayesian learning framework initially proposed by West et al. [56]. This framework utilizes the prior distribution of system states, which is quantified based on historical data. Many studies have contributed to the development of this framework. For example, Abernathy et al. [2] and Diaconis and Ylvisaker [16] summarized the conjugate priors for various distributions in the exponential family. Further, Chen et al. [12], Lavine et al. [31], and Soyer and Zhang [47] developed the Bayesian sequential learning theory for estimating the posterior distribution of system states based on new data collected over time.

Our work directly follows the work initially developed by Berry and West [5] and Berry et al. [6], which presented a state-of-the-art Bayesian forecasting framework. This framework can be used to perform many-item, multi-step-ahead count-valued time series forecasting. The authors demonstrated improved forecast accuracy on multiple metrics and illustrated the benefits of full probabilistic models for forecast accuracy evaluation and comparison [54]. To capture nonlinear relationships and changing patterns over time, advanced methods such as embedding generalized linear models within a Bayesian forecasting framework may be necessary. These Bayesian generalized linear models provide dynamic extensions to the standard generalized linear models [55].

## MDS data processing

**Fig. 1:**
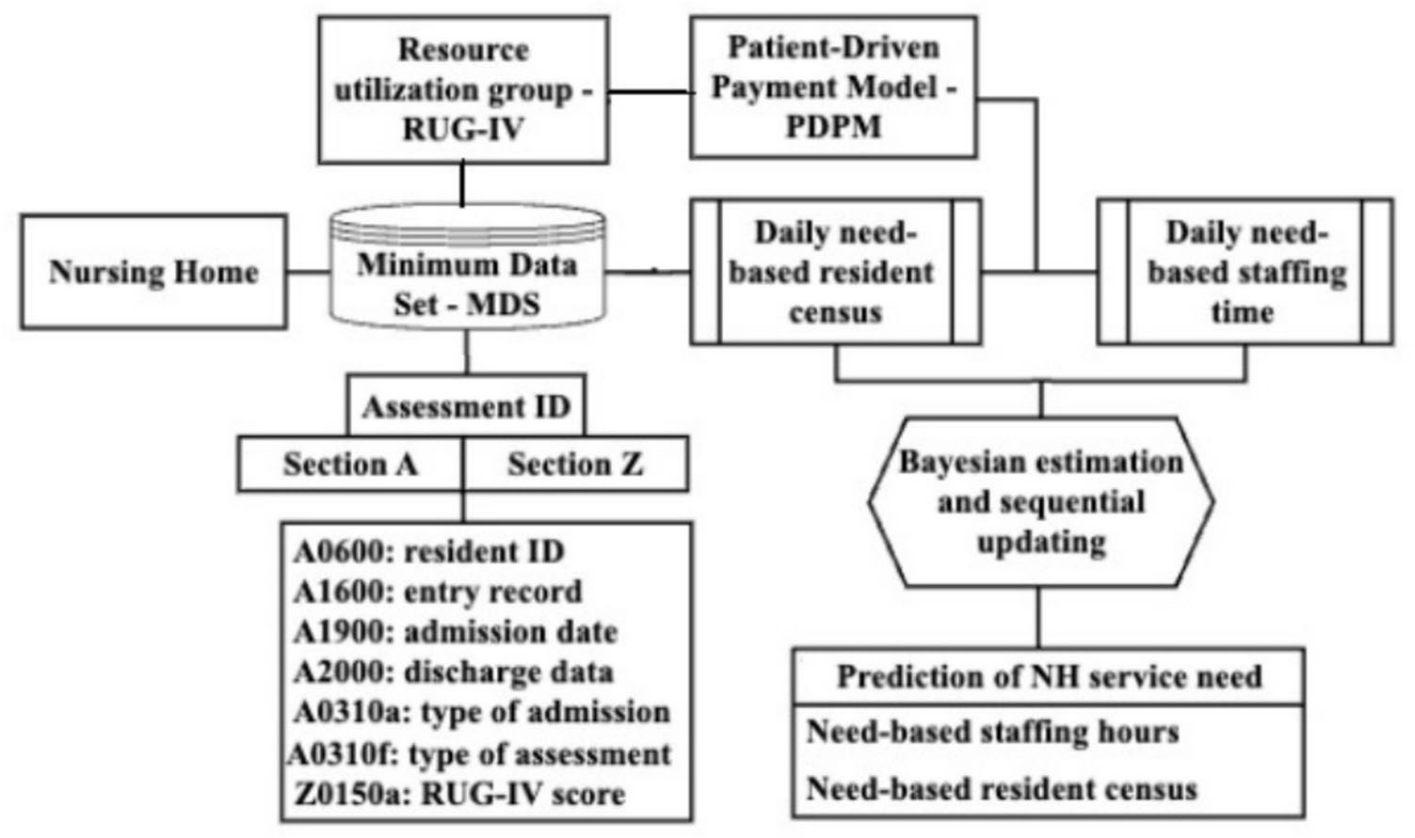
Data processing pipeline to generate the time series data for case-mix service need

Figure 1 provides a schematic overview of our data processing pipeline for quantifying and forecasting service need of a NH with resident mix. Our focus is processing MDS data to capture individual resident’s clinical assessment over time. The MDS 3.0 data contains 21 sections that keep track of a wide range of NH residents’ health and functional conditions. The dataset also records each resident’s admission and discharge information. By analyzing these data, we can generate a better understanding of facility-wide NH service need over time.

We now provide details on how we process MDS 3.0 data. First, we extracted the data in section A *Identification Information* and section Z *Assessment Administration* from each resident’s report. We then utilized resident admission and discharge records to establish the acuity category of each resident, which yields the resident census on any given day. We next used the RUG-IV classification system to assign residents to 48 RUGs. We use the National Provider Identifiers (NPIs) to extract all resident assessment IDs for the studied NH from the A0100a data field in section A. We merged the data from sections A and Z using the assessment IDs as the key. After the merging, each row of the data frame contains the information of one assessment entry. The data frame has 63 columns. Each resident has a unique de-identified resident ID, recorded in field A0600 of section A.

We then removed duplicate entries of the same acuity assessment in each record to clean the data. In addition, there are two types of missing data: missing discharge date and missing assessment summary. We followed CMS-recommended steps to deal with patient records with missing discharge date [50]. Specifically, we used the A0310 data field in section A to identify the assessment type (annual assessment, quarterly assessment, death in facility, or significant change) and calculated the resident’s length of stay at the facility. For records with missing assessment, we filled in the misses with the most recent assessment results.

We next exacted each individually tracked assessment record with the de-identified resident ID as the key and generate the RUG for each resident over time. Based on each resident’s assessment results and RUG classification, we subsequently followed the PDPM to specify their CMS-recommended need-based staff time on any day. Finally, we aggregated the information about each resident’s acuity classification and need-based staff time determination to generate the daily acute group-specific resident volume and caregiver type-specific staff time for an NH. As a result, we generate multi-dimensional time series for daily service need measures. Note that our focus in the data generation has been adequate service needs based on acuity category. The forecasting approach we are about to propose is indifferent of the staff time being adequate or not and how resident census is constructed.

## A model-based bayesian forecasting approach

### Resident volume modeling

Accurate modeling of NH resident census is crucial to NH workforce planning. Our first model is thus designed to handle non-negative discrete time-series data with potentially excessive zeroes.

Considering residents in an NH, which fall in *G* distinct groups, we model the daily resident volume over time of any group $g, g=1,..., G$. We denote the observed resident volume of group *g* on day *t* as $z_{g,t}$. Essentially, we deal with multi-dimensional NH resident volume data. We present a discrete census mixture model (DCMM) [5, 6] for modeling $z_{g,t}$, which possibly contains excessive zeros, as:

1 $$\begin{aligned} z_{g,t} \vert x_{g,t}= & \Biggl \{\begin{array}{ll} 0 & \text {if} \quad x_{g,t}=0\\ \zeta _{g,t},& \text { if } x_{g,t}=1; \end{array}\end{aligned}$$

2 $$\begin{aligned} x_{g,t}\sim & \text {Ber}(p_{g,t}),\quad \zeta _{g,t}\sim \text {Poi}_+(\eta _{g,t});\end{aligned}$$

3 $$\begin{aligned} \text {logit}(p_{g,t})= & (\boldsymbol{\tilde{F}}^{\text {D}}_{g,t})^{\prime }\boldsymbol{\tilde{\theta }}^{\text {D}}_{g,t}, \quad \text {log}(\eta _{g,t})= (\boldsymbol{F}^{\text {D}}_{g,t})^{\prime }\boldsymbol{\theta }^{\text {D}}_{g,t};\end{aligned}$$

4 $$\begin{aligned} \begin{pmatrix} \boldsymbol{\tilde{\theta }}^{\text {D}}_{g,t}\\ \boldsymbol{\theta }^{\text {D}}_{g,t}\end{pmatrix}= & \boldsymbol{G}^{\text {D}}_{g,t} \begin{pmatrix} \boldsymbol{\tilde{\theta }}^{\text {D}}_{g,t-1}\\ \boldsymbol{\theta }^{\text {D}}_{g,t-1}\end{pmatrix}+\boldsymbol{\omega }^{\text {D}}_{g,t}, \end{aligned}$$

where we introduce a time-varying Bernoulli latent variable $x_{g,t} \sim \text {Ber}(p_{g,t})$ in Eq. 1 to separate zero and positive census counts in group *g* on day *t*. The zero count data often occurs when all residents in some group have been discharged and no new admissions take place. We use $x_{g,t}=0$ to denote that the situation of zero data occurs in group *g* and use $1-p_{g,t}$ to quantify the probability of such situation. Otherwise, if $x_{g,t}=1$, we will observe positive census counts following a truncated-zero discrete distribution (e.g., truncated-zero Poisson distribution) in Eq. 2 with time-varying intensity $\eta _{g,t}$. We associate $p_{g,t}$ and $\eta _{g,t}$ in Eq. 3 by incorporating potential influencing factors represented by column vectors $\boldsymbol{\tilde{F}}^{\text {D}}_{g,t}$ and $\boldsymbol{F}^{\text {D}}_{g,t}$ with time-varying effects quantified by column vectors $\boldsymbol{\tilde{\theta }}^{\text {D}}_{g,t}$ and $\boldsymbol{\theta }^{\text {D}}_{g,t}$, respectively. Superscript notation “D” implies the discrete-scale modeling.

To promptly capture the evolving patterns of the census data over time, we treat $\boldsymbol{\tilde{\theta }}^{\text {D}}_{g,t}$ and $\boldsymbol{\theta }^{\text {D}}_{g,t}$ as latent state variables. They reflect the intensity of resident volume of group *g* observed at time *t*. We model their dynamics at two consecutive time-stamps (i.e., *t* and $t-1$) in Eq. 4 with state evolution matrix $\boldsymbol{G}^{\text {D}}_{g,t}$, where $\boldsymbol{\omega }^{\text {D}}_{g,t}$ is a Gaussian random vector capturing the error term at time *t* for group *g* with mean $\boldsymbol{0}$ and variance matrix $\boldsymbol{W}^{\text {D}}_{g,t}$. It is used to capture the degree of temporal variability in the latent state transitions over time. In practice, a positive entry in $\boldsymbol{G}^{\text {D}}_{g,t}$ indicates that there is an increasing intensity of observing resident volume of resident group *g* at time *t* due to the corresponding latent variable, as compared to that at the previous time point $t-1$, and vice versus. Meanwhile, if the volume data exhibits significant fluctuations, indicating that the underlying latent states change substantially over time, a larger variance is specified for the noise term. Conversely, if the latent state evolves more smoothly, a smaller variance is used.

### Staff time modeling

When making staffing and scheduling decisions for each type of caregivers in a NH, it is more informative and precise to measure staff time with a continuous scale. This provides sufficient granularity in NH’s operations management practice. Our second model is thus tailored to handle continuous time-series data.

Again consider a NH, which comprises residents in *G* distinct group *g*, $g = 1, \ldots , G$. We denote the observed facility-wide staff time needed on day *t* of type-*r* caregivers as $d_{r,t}$ for type-*r* caregiver. Due to lack of actual staff time data from local NHs, we utilize the PDPM as a reference on CMS-recommended need-based staff time for each caregiver type to quantify the staff time. That is, $d_{r,t} = \sum _{g \in G} \lambda _{r,g} z_{g,t}$, where $\lambda _{r,g}$ is the daily PDPM-specified staff time of type-*r* caregiver for one resident in group *g* and $z_{g,t}$, as defined earlier, is the resident volume of group *g* on day *t*. Essentially, we deal with multi-dimensional NH staff time data. We present a dynamical generalized linear model (DGLM) [5, 6] for modeling $\log {d_{r,t}}$, which meets the DGLM modeling assumptions, as:

5 $$\begin{aligned} \log {d_{r,t}}\sim & \text {N}(\mu _{r,t},\sigma ^2_{r,t});\end{aligned}$$

6 $$\begin{aligned} \mu _{r,t}= & (\boldsymbol{\tilde{F}}_{r,t}^{\text {C}})^{\prime }\boldsymbol{\tilde{\theta }}^\text {C}_{r,t}, \quad \log (\sigma ^2_{r,t})=(\boldsymbol{F}_{r,t}^{\text {C}})^{\prime }\boldsymbol{\theta }^\text {C}_{r,t};\end{aligned}$$

7 $$\begin{aligned} \begin{pmatrix} \boldsymbol{\tilde{\theta }}_{r,t}^{\text {C}} \\ \boldsymbol{\theta }^{\text {C}}_{r,t} \end{pmatrix}= & \boldsymbol{G}^{\text {C}}_{r,t}\begin{pmatrix}\boldsymbol{\tilde{\theta }}^{\text {C}}_{r,t-1}\\ \boldsymbol{\theta }^{\text {C}}_{r,t-1} \end{pmatrix} +\boldsymbol{\omega }^{\text {C}}_{r,t}, \end{aligned}$$

where we capture the log scale of the staff time with a Gaussian distribution with caregiver type-specific and time-varying parameters in Eq. 5. A lognormal distribution is reflective of the observed staff time with most observations being around the standardized value but a small portion of observations being long due to unexpected service duties. To further associate the mean and variance parameters with potential influencing factors, we incorporate vectors $\boldsymbol{\tilde{F}}^{\text {C}}_{r,t}$ and $\boldsymbol{{F}}^{\text {C}}_{r,t}$ with their time-varying effects characterized by latent state vectors $\boldsymbol{\tilde{\theta }}^{\text {C}}_{r,t}$ and $\boldsymbol{{\theta }}^{\text {C}}_{r,t}$, respectively; see Eq. 6. Similar to the acuity-specific resident volume, we again model the temporal dynamics of latent state variables over two consecutive days with random error vector $\boldsymbol{\omega }^{\text {C}}_{r,t}$; see Eq. 7. Superscript notation “C” implies the continuous-scale modeling.

### Incorporation of shared information

In reality, there often exists correlations between the resident volume (or staff time) of different acuity categories (or caregiver types). For instance, an NH may recently admit a set of post-acute care residents belonging to multiple groups (i.e., acuity categories) from the same emergency care event. Moreover, when caring for a resident who suffers from multiple chronic conditions and functional limitations, RNs or LPNs need to periodically assess the resident’s health condition and care needs while CNAs need to provide restorative care to maintain or delay the further deterioration of the resident’s functional limitations. The staff time needed among different caregiver types may therefore be correlated to a certain extent over time as well. Incorporating the shared information into the acuity category-specific (or caregiver type-specific) resident volume (staff time) prediction model presented earlier may further improve the prediction.

With the incorporation of the shared information, the augmented covariate vectors for acuity group *g* become$$\boldsymbol{\tilde{F}}^{\mathrm{S-D}}_{g,t}=\begin{pmatrix}\boldsymbol{\tilde{F}}^D_{g,t}\\\boldsymbol{\tilde{h}}^{\mathrm{S-D}}_{t}\end{pmatrix}]$$ and $$\boldsymbol{F}^{\mathrm{S-D}}_{g,t}=\begin{pmatrix}\boldsymbol{F}^D_{g,t}\\\boldsymbol{h}^{\mathrm{S-D}}_{t}\end{pmatrix}$$, respectively. Here $\boldsymbol{\tilde{F}}^D_{g,t}$ and $\boldsymbol{F}^D_{g,t}$ contain constants and acuity group specific predictors. The shared covariate vectors $\boldsymbol{\tilde{h}}^{\text {S-D}}_{t}$ and $\boldsymbol{h}^{\text {S-D}}_{t}$ are common to all series representing the shared information among correlated acuity groups. Accordingly, the corresponding augmented state vectors after incorporating the shared information become $$\boldsymbol{\tilde{\theta}}^{\mathrm{S-D}}_{g,t}=\begin{pmatrix}\boldsymbol{\tilde{\theta}}^D_{g,t}\\\boldsymbol{\tilde{\gamma}}^{\mathrm{S-D}}_{g,t} \end{pmatrix}$$ and $$\boldsymbol{\theta}^{\mathrm{S-D}}_{g,t}=\begin{pmatrix} \boldsymbol{\theta}^D_{g,t}\\\boldsymbol{\gamma}^{\mathrm{S-D}}_{g,t}\end{pmatrix}$$. Each series has its own state vector components $\boldsymbol{\tilde{\gamma }}^{\text {S-D}}_{g,t}$ and $\boldsymbol{\gamma }^{\text {S-D}}_{g,t}$ so that the impacts of common factors are category-specific and time-varying. 

Similarly, in the continuous service need prediction model, with the incorporation of the shared information, i.e., staffing time of correlated caregiver types, the augmented covariates for type-*r* caregivers become $\boldsymbol{\tilde{F}}^{\text {S-C}}_{r,t}$ and $\boldsymbol{F}^{\text {S-C}}_{r,t}$ with augmented state variables $\boldsymbol{\tilde{\theta }}^{\text {S-C}}_{r,t}$ and $\boldsymbol{\theta }^{\text {S-C}}_{r,t}$.

### Bayesian estimation and sequential model updating

Given the proposed discrete and continuous-valued service need models above, we in this section propose a unified Bayesian estimation procedure that allows convenient sequential updating of the model parameters. The intention of unifying both models under the same form is to explain the Bayesian estimation procedure in a general and unified fashion. Specifically, we denote $y_t$ to be the observed quantity at time *t* (i.e., resident volume $z_{g,t}$ or staff time $d_{r,t}$).

We unify Eqs. (2) and (5) under the standard exponential family density form as:

8 $$\begin{aligned} f(y_t\vert \boldsymbol{\phi }_t) = \exp [\boldsymbol{c}(\boldsymbol{\phi }_t)^{\prime }\boldsymbol{T}(y_t) -a( \boldsymbol{\phi }_t)]b(y_t), \end{aligned}$$

where $\boldsymbol{\phi }_t$ represents a collection of unknown parameters, e.g., $\phi _t=p_{g,t}$ or $\phi _t=\eta _{g,t}$ in Eq. 2 and $\boldsymbol{\phi }_t=\{\mu _{r,t}, \sigma ^2_{r,t} \}$ in Eq. 5. In the discrete case, Bernoulli distribution in Eq. 2 is first used to separate zero versus positive census counts data, and zero-truncated Poisson distribution is further used to model positive census counts data. Specifically, when separating zero versus positive census counts $z_{g,t}$, $y_t=x_{g,t}$. In other words, when $z_{g,t}>0$, we have $y_t=1$; When $z_{g,t}=0$, we have $y_t=0$. Bernoulli distribution in Eq. 2 can be represented as Eq. 8 with $\phi _t=p_{g,t}$, $T(y_t)=y_t$, $c(\phi _t)=\log (\frac{p_{g,t}}{1-p_{g,t}})$, $a(\phi _t)=-\log (1-p_{g,t})$ and $b(y_t)=1$. After separating zero and positive counts data, zero-truncated Poisson in Eq. 2 is further used to model positive counts data, i.e., $y_t=\zeta _{g,t}=z_{g,t}$ for $z_{g,t}>0$. The zero-truncated Poisson can be represented as Eq. 8 with $\phi _t=\eta _{g,t}$, $T(y_t)=y_t$, $c(\phi _t)=\log (\eta _{g,t})$, $a(\phi _t)=\log (\exp {\eta _{g,t}}-1)$ and $b(y_t)=\frac{1}{y_t!}$. In the continuous case, we have $y_t=\log (d_{r,t})$, and normal density can be further represented as Eq. 8 with $\boldsymbol{\phi }_t=[\mu _{r,t},\sigma ^2_{r,t}]^{\prime }$, $\boldsymbol{T}(y_t)=[y_t,y_t^2]^{\prime }$, $\boldsymbol{c}(\boldsymbol{\phi }_t)=[\frac{\mu _{r,t}}{\sigma ^2_{r,t}},\frac{-1}{2\sigma ^2_{r,t}}]^{\prime }$, $a(\phi _t)=\frac{\mu ^2_{r,t}}{2\sigma ^2_{r,t}}+\frac{1}{2}\log (\sigma ^2_{r,t})$ and $b(y_t)=\frac{1}{\sqrt{2\pi }}$.

Based on the historical service need data from the beginning of the study period to time $t-1$, we denote $\boldsymbol{\mathcal {D}}_1^{t-1}= \{y_1,\ldots , y_{t-1} \}$, model parameters $\boldsymbol{\phi }_t$ can be sequentially updated under a coherent Bayesian framework as

9 $$\begin{aligned} \pi _t(\boldsymbol{\phi }_t \vert \boldsymbol{\mathcal {D}}_1^{t})\varpropto f(y_t\vert \boldsymbol{\phi }_t) \cdot \pi _{t-1}(\boldsymbol{\phi }_t \vert \boldsymbol{\mathcal {D}}_1^{t-1}), \end{aligned}$$

where $\boldsymbol{\mathcal {D}}_1^{t}$ represents all the service need data observed/computed until time *t*, i.e., $\boldsymbol{\mathcal {D}}_1^{t}=\boldsymbol{\mathcal {D}}_1^{t-1} \bigcup y_t$. $\pi _{t-1}(\cdot )$ and $\pi _t(\cdot )$ are the prior and posterior densities for $\boldsymbol{\phi }_t$ at time *t*, respectively.

To ensure both densities are in the same distribution form, we specify a conjugate prior for $\boldsymbol{\phi }_t$ with parameter vector $\boldsymbol{\alpha }_{t-1}$. Subscript $t-1$ is used to emphasize that the prior is elicited based on data sequentially attainable up to time $t-1$. The prior elicitation procedure is described as follows. First, to determine prior vector $\boldsymbol{\alpha }_{t-1}$, we specify latent state vector $\boldsymbol{\theta }_t$ with prior mean vector $\boldsymbol{m}^{\theta _t}_{t-1}=\mathbb {E}[\boldsymbol{\theta }_t \vert \boldsymbol{\mathcal {D}}_1^{t-1}]$ and prior variance matrix $\boldsymbol{V}^{\theta _t}_{t-1}=\mathbb {V}[\boldsymbol{\theta }_t \vert \boldsymbol{\mathcal {D}}_1^{t-1}]$. In the discrete model, latent state vector $\boldsymbol{\theta }_t$ refers to $\boldsymbol{\theta }_{g,t}^{\text {D}}$ and $\boldsymbol{\tilde{\theta }}^{\text {D}}_{g,t}$. In the continuous model, latent state vector $\boldsymbol{\theta }_t$ refers to $\boldsymbol{\theta }_{r,t}^{\text {C}}$ and $\boldsymbol{\tilde{\theta }}^{\text {C}}_{r,t}$. The prior mean and variance of $\boldsymbol{\theta }_t$ can be essentially computed from the posterior mean vector $\boldsymbol{m}^{\theta _{t-1}}_{t-1}=\mathbb {E}[\boldsymbol{\theta }_{t-1} \vert \boldsymbol{\mathcal {D}}_1^{t-1}]$ and variance matrix $\boldsymbol{V}^{\theta _{t-1}}_{t-1}=\mathbb {V}[\boldsymbol{\theta }_{t-1} \vert \boldsymbol{\mathcal {D}}_1^{t-1}]$ of $\boldsymbol{\theta }_{t-1}$ using the latent states evolution equations, i.e., Eqs. 4 and 7 as

10 $$\begin{aligned} \boldsymbol{m}^{\theta _{t}}_{t-1}= & \boldsymbol{G}_{t}\boldsymbol{m}^{\theta _{t-1}}_{t-1}, \end{aligned}$$

11 $$\begin{aligned} \boldsymbol{V}^{\theta _t}_{t-1}= & \boldsymbol{G}_{t}\boldsymbol{V}_{t-1}^{\theta _{t-1}}\boldsymbol{G}_{t}'+\boldsymbol{W}_{t}, \end{aligned}$$

where in the discrete model, latent state matrix $\boldsymbol{G}_{t}$ refers to $\boldsymbol{G}_{g,t}^{\text {D}}$ and variance matrix $\boldsymbol{W}_{t}$ refers to $\boldsymbol{W}_{g,t}^{\text {D}}$ of $\boldsymbol{\omega }_{g,t}^{\text {D}}$. In the continuous model, matrix $\boldsymbol{G}_t$ refers to $\boldsymbol{G}_{r,t}^{\text {C}}$, and matrix $\boldsymbol{W}_{t}$ refers to $\boldsymbol{W}_{r,t}^{\mathrm{C}}$ of $\boldsymbol{\omega}_{r,t}^{\mathrm{C}}$.

Second, based on the existing link functions between latent states and parameters (i.e., Eqs. 3 and 6), the prior mean vector and variance matrix of $\boldsymbol{\phi }_t$, namely $\boldsymbol{m}^{\phi _t}_{t-1}=\mathbb {E}[\Delta (\boldsymbol{\phi }_t) \vert \boldsymbol{\mathcal {D}}_1^{t-1}]$ and prior variance $\boldsymbol{V}^{\phi _t}_{t-1}=\mathbb {V}[\Delta (\boldsymbol{\phi }_t) \vert \boldsymbol{\mathcal {D}}_1^{t-1}]$ can be computed as

12 $$\begin{aligned} \boldsymbol{m}^{\phi _t}_{t-1}=\boldsymbol{F}'_{t}\boldsymbol{m}^{\theta _{t}}_{t-1},\quad \boldsymbol{V}^{\phi _t}_{t-1}=\boldsymbol{F}'_{t}\boldsymbol{V}^{\theta _{t}}_{t-1}\boldsymbol{F}_{t}, \end{aligned}$$

where in the discrete model, $\Delta (\cdot )$ functions refer to the logit and log functions for $p_{g,t}$ and $\eta _{g,t}$ respectively. Covariate vector $\boldsymbol{F}_{t}$ refers to $\tilde{\boldsymbol{F}}_{g,t}^{\text {D}}$ and $\boldsymbol{F}_{g,t}^{\text {D}}$. In the continuous model, $\boldsymbol{F}_t$ refers to $\tilde{\boldsymbol{F}}_{r,t}^{\text {C}}$ and $\boldsymbol{F}_{r,t}^{\text {C}}$, and vector $\boldsymbol{\omega }_{t}$ refers to $\boldsymbol{\omega }_{r,t}^{\text {C}}$ with variance matrix $\boldsymbol{W}_{r,t}^{\text {C}}$. With the prior mean vector $\boldsymbol{m}^{\phi _t}_{t-1}$ and variance matrix $\boldsymbol{V}^{\phi _t}_{t-1}$ calculated in Eq. 12, prior parameters $\boldsymbol{\alpha }_{t-1}$ can be obtained using a moments matching method.

To ensure convenient sequential updating in Eq. 9, conjugate priors are specified for $\boldsymbol{\alpha }_t$. In particular, a Bernoulli prior and a Gamma prior are specified for $p_{g, t}$ and $\eta _{g,t}$, respectively, in the discrete model. A normal-inverse Gamma prior is specified for the $(\mu _{r,t},\sigma ^2_{r,t})$ pair in the continuous model. It is noticed that $\boldsymbol{\alpha }_{t-1}$ are prior parameters containing all information till time $t-1$ and $\boldsymbol{\alpha }_t$ are posterior parameters containing all information till time *t* based on Eq. 9.

With Bayesian sequential updating via Eq. (9), the updated posterior mean vector and variance matrix of the estimated parameters $\phi _t$ based on data attained up to time *t* become $\boldsymbol{m}^{\phi _t}_{t}$ and $\boldsymbol{V}^{\phi _t}_{t}$. Based on the inverse of existing link functions between latent states and parameters (i.e., Eqs. 3 and 6), the posterior mean and variance of latent states $\boldsymbol{\theta }_t$, namely $\boldsymbol{m}^{\theta _t}_{t}$ and $\boldsymbol{V}^{\theta _t}_{t}$, can be updated as [5, 6]

13 $$\begin{aligned} \boldsymbol{m}^{\theta _t}_{t}= & \boldsymbol{m}^{\theta _t}_{t-1}+\boldsymbol{V}^{\theta _t}_{t-1}\boldsymbol{F}_t \left( \boldsymbol{m}^{\phi _t}_{t}-\boldsymbol{m}^{\phi _t}_{t-1}\right) /\boldsymbol{V}^{\phi _t}_{t-1} ,\end{aligned}$$

14 $$\begin{aligned} \boldsymbol{V}^{\theta _t}_{t}= & \boldsymbol{V}^{\theta _t}_{t-1}-\boldsymbol{V}^{\theta _t}_{t-1}\boldsymbol{F}_t\boldsymbol{F}_t'\boldsymbol{V}^{\theta _t\prime }_{t-1}\left( 1-\frac{\boldsymbol{V}^{\phi _t}_{t}}{\boldsymbol{V}^{\phi _t}_{t-1}}\right) /\boldsymbol{V}^{\phi _t}_{t-1}. \end{aligned}$$

With vector $\boldsymbol{m}^{\theta _t}_{t}$ and matrix $\boldsymbol{V}^{\theta _t}_{t}$ computed by Eqs. 13) and 14, they can be used to update prior mean and variance of latent states $\boldsymbol{\theta }_{t+1}$ at time $t+1$ via Eqs. (10) and (11) and further update the prior mean and variance of parameters $\boldsymbol{\phi }_{t+1}$ at time $t+1$ in a recursive manner via Eq. (12). In addition, to incorporate shared information, the Bayesian estimation and sequential model updating procedures will remain the same by replacing latent state vectors $\boldsymbol{\tilde{\theta }}^{\text {D}}_{g,t},\boldsymbol{\theta }^{\text {D}}_{g,t}$,$\boldsymbol{\tilde{\theta }}^{\text {C}}_{r,t},\boldsymbol{\theta }^{\text {C}}_{r,t}$ with $\boldsymbol{\tilde{\theta }}^{\text {S-D}}_{g,t},\boldsymbol{\theta }^{\text {S-D}}_{g,t}$,$\boldsymbol{\tilde{\theta }}^{\text {S-C}}_{r,t},\boldsymbol{\theta }^{\text {S-C}}_{r,t}$ and further replacing influencing factors represented by vectors $\boldsymbol{\tilde{F}}^{\text {D}}_{g,t},\boldsymbol{F}^{\text {D}}_{g,t}$,$\boldsymbol{\tilde{F}}^{\text {C}}_{r,t},\boldsymbol{F}^{\text {C}}_{r,t}$ with vectors $\boldsymbol{\tilde{F}}^{\text {S-D}}_{g,t},\boldsymbol{F}^{\text {S-D}}_{g,t}$,$\boldsymbol{\tilde{F}}^{\text {S-C}}_{r,t},\boldsymbol{F}^{\text {S-C}}_{r,t}$ as defined previously.

Unlike employing Markov chain Monte Carlo (MCMC) sampling techniques (or other Bayesian sampling techniques) to jointly estimate unknown parameters and latent states, our model updating procedure described above essentially takes advantage of the model structure and considers the moment matching method to calculate the posterior mean and variance for both unknown parameters and latent states. The combination of such moment matching method and Bayesian updating principle will naturally facilitate the sequential updating of the unknown parameters and latent states, which bypasses the potential computational complexity of a full Bayesian sampling approach. The pseudo-code for updating the prediction model is summarized as follows. 
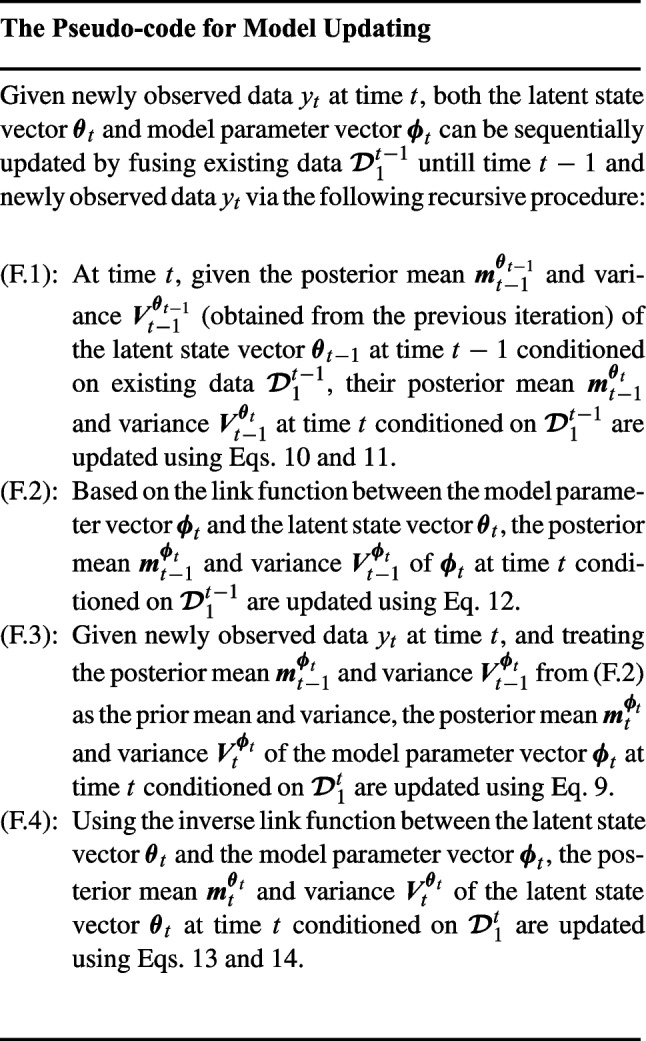


### Multi-step forecasting with rolling window

In this section, we embed the idea of Bayesian estimation and sequential updating in Section 4.4 in a multi-step forecasting framework with rolling window along a forecasting horizon. To provide effectively multi-step forecasting along a horizon, existing work mainly consists of two strategies: recursive and direct forecasting [49]. Recursive forecasting involves estimating a single time series model and using it to compute each forecast by utilizing previous forecasts. However, this strategy is known to be biased when the underlying model is nonlinear. On the other hand, direct forecasting involves estimating a separate time series model for each forecasting horizon and computing forecasts based only on attained data. This strategy is less biased than recursive forecasting, but it has higher variance due to the use of fewer observations when estimating the model, especially for longer forecast horizons.

In light of the bias-variance trade-off associated with the choice between a directive and recursive forecasting scheme, we investigated both strategies for our forecasting tasks [13]. We considered a forecasting process on the quantity of interest (i.e., $y_t$ unified in Section 4.4) with multiple time points of updating the prediction models and a rolling forecasting time window. We constructed separate models at each time step, and each model could use the predictions made by the model at prior time steps as input values. For example, at time *t*, we look ahead over future *K* time points based on the current information $\boldsymbol{\mathcal {D}}_1^t$. The forecasting for the immediate future time point will reflect the predictive distribution at *t*, which is given by

15 $$\begin{aligned} p(y_{t+1}\vert \boldsymbol{\mathcal {D}}_1^{t} )=F_{t}(\boldsymbol{\mathcal {D}}_1^{t}). \end{aligned}$$

For the $k^{th} (k\ge 2)$ future time points, the predictive distribution is characterized as

16 $$\begin{aligned} p(y_{t+k}\vert \boldsymbol{\mathcal {D}}_1^{t+k-2},\hat{y}_{t+k-1} )=F_{t+k-1}(\boldsymbol{\mathcal {D}}_1^{t+k-2},\hat{y}_{t+k-1}). \end{aligned}$$

Note that the length of the forecast window (between two consecutive time points) and the duration of the forecast horizon can be specified by the NH itself. In a nutshell, we update both the latent state vector and model parameter vector at time *t* recursively based on the integration of historical data up to *t*-1, and newly observed data at *t*. The updated posterior mean and variance of the latent state vector serve as the input of the next iteration, enabling recursive update as future data become available. Based on the estimated latent states and parameter vectors, we obtain estimated predictive distributions for both service need quantities. To generate point forecasts, we sample extensively from these estimated predictive distributions and use the sample medians as the point estimates as forecast values at *t*.

**Table 1:** Summary of organization, resident, and staffing characteristics of the three selected NHs.*

NH characteristics	Categories	NH1	NH2	NH3
Organization	Number of Beds	112	24	86
	Occupancy rate	42.1%	26.5%	66.7%
	Long-term rating	5	Not available	5
	Short-term rating	5	5	Not available
Resident	Average number of residents	47.2	6.3	57.4
	Average length of stay (days)	34.8	24.2	34.1
	Median age	81	82	86
	Number of RUGs being served	27	18	18
	# of RUGs with volatile resident volume	26	13	8
	# of RUGs in need of extended staffing	16	12	9
Staffing	RN** hprd***	1.6	2.8	0.9
	LPN** hprd	1.9	3.1	0.3
	CNA** hprd	2.6	4.8	3.1

* Information accessed in Dec 2022

** RN, registered nurse; LPN, licensed practical nurses; CNA, certified nursing assistants

*** hprd, hours per resident day

## Experiments and discussions

### Test NH selection

To create our case study datasets, we used the information available on the CMS Nursing Home Compare website [32], and chose three licensed NHs in the state of Indiana (termed NH1, NH2, and NH3). All three NHs are reputable and known for providing high-quality care (i.e., CMS rating of 1 star - 5 stars with 5 being the highest [30]). Basic information from late 2022 on the three is summarized in Table 1.

We selected these three NHs for their diverse need and service patterns. The service in the NHs is classified into short-term care and long-term care. Short-term care usually involves short-term rehabilitation for residents who have been released from the hospital following a serious illness, injury, or surgery, and need more time to recover before safely returning home. These residents typically need extended medical care, nursing care, and therapies for only a few weeks. In contrast, long-term care residents mainly have medical needs and require round-the-clock assistance and monitoring through the rest of their lives [42].

NH1 offers both long-term and short-term care services, and it has more beds than the other two NHs. NH2 only provides short-term care with fewer beds and lower bed occupancy. NH3 only offers long-term care and typically has higher bed occupancy. There are also significant differences in resident population and length of stay among the three NHs. Notably, NH2 has fewer residents and shorter stay duration on average whereas NH3 is just the opposite.

The three NHs primarily provide care to elderly residents aged 65 and above, with slight variation in resident’s median age. Among the three, NH1 had the most diverse cohort with residents assigned to 27 RUGs in total. With an Augmented Dickey–Fuller test [19], we found that the proportion of RUGs in NH1 that had non-stationary resident volume was greater than the other two NHs. We also found that the proportion of RUGs in NH1 where residents were in need of extended staffing (i.e., more than 1.5 hours of RN time each day) was about the same as the other two.

The staffing level differs across the three NHs. NH2, due to its focus on short-term residents, had the highest staffing level (measured by staff hours per resident day) by all three caregiver types, among the three NHs On the other hand, NH3, with its focus on long-term residents, had the lowest staffing level. NH1, for providing both short- and long-term care, falls in between NH2 and NH3, in terms of staffing level.

**Fig. 2:**
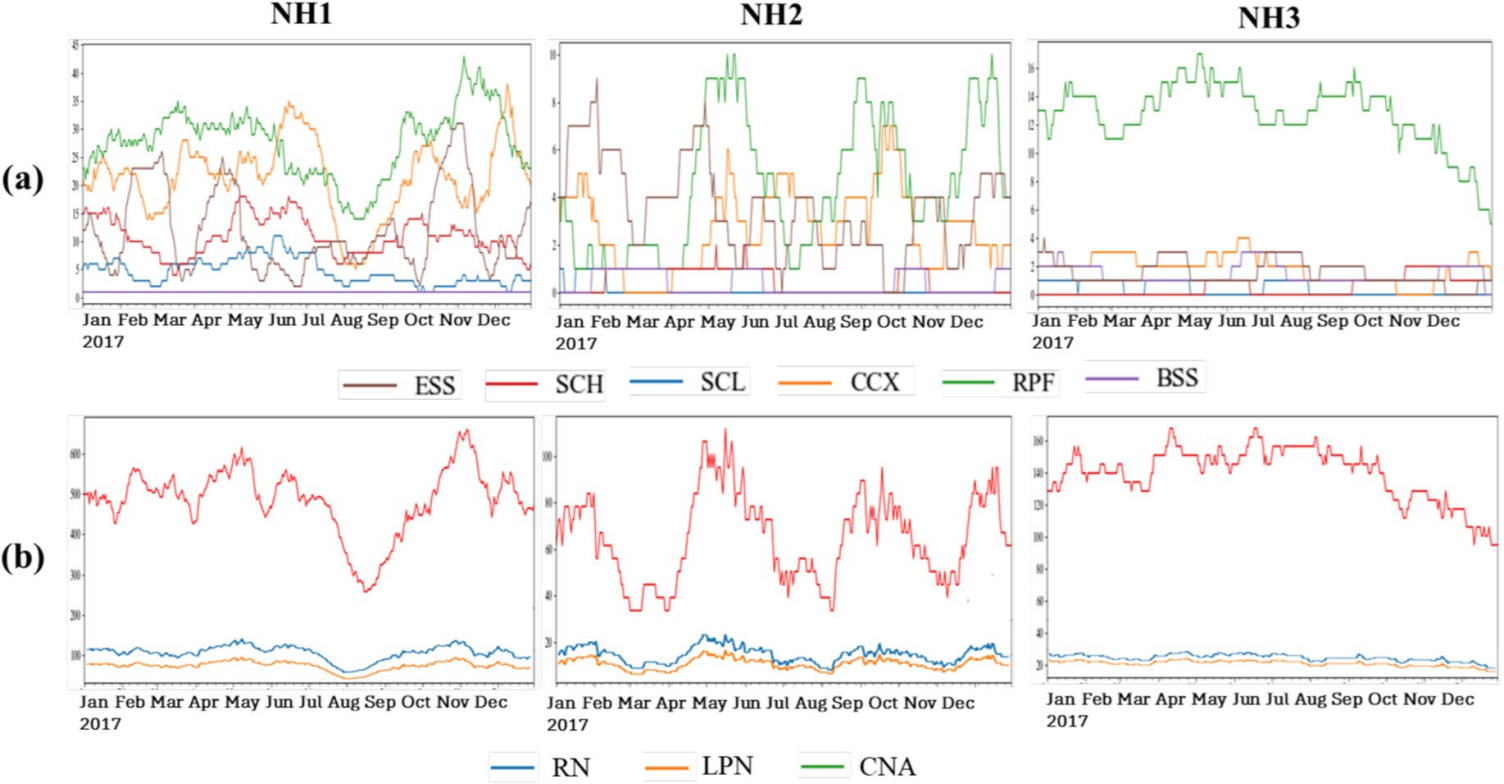
Daily resident volume and staff time by group for each NH (each subfigure column). Upper row: resident volume of each of the 6 acuity categories; Lower row: staff time of each of the 3 caregiver types)

**Fig. 3:**
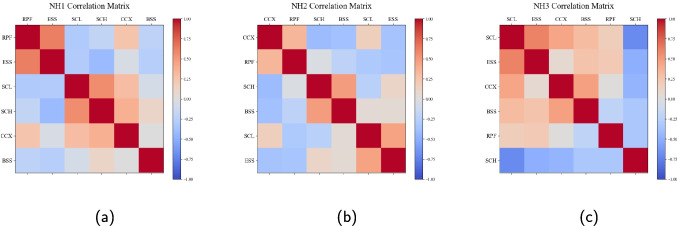
Correlation matrices. Each correlation coefficient is derived from changes in the resident volumes in a pair of acuity categories

### Preliminary analyses

We extracted one-year MDS data from the three selected NHs between January 2017 and December 2017. It is worth noting that information on facility characteristics is not well kept in online sources over time. As a result, we had to take the information from 2022, as shown in Table 1. To ensure that the 2022 facility information about the three selected NHs still provides insights into them and their care in 2017, we checked the general trend of organizational characteristics (e.g., bed capacity, occupancy rate, etc.) from 2017 – 2022 [32]. We did not find significant changes over the years.

Following the data processing procedure outlined in Section 3, we curated the daily resident volume data for each acuity category. We subsequently calculated the facility-wide staff time (in hours) for the three caregiver types (i.e., RN, LPN, and CNA). The resultant resident volume and staff time data are presented in Fig. 2 with the three subfigures in each row depicting the time series for the three NHs. To align with our numerical studies which only focused on six non-rehab-focused major categories (with a higher level of caregiving diversity), we only present the resident volume data for those six categories: (1) extensive care (ESS), (2) special care high (SCH), (3) special care low (SCL), (4) clinical complex (CCX), (5) behavioral symptoms (BSS), and (6) reduced physical function (RPF).

We further used hierarchical clustering [36] to assess correlations among groups to verify the need of incorporating shared information. Figure 3 illustrates the correlations among acuity categories. Our results, as shown in Fig. 3a, suggest strongly positive correlations among the SCL, SCH, and CCX categories in NH1. On the other hand, our results, as shown in Fig. 3c, suggest strongly positive correlations between the BSS, CCX, and SCL categories in NH3 but every category above is negatively correlated with SCH. NH2 is found to be between NH1 and NH3 in terms of the correlation results. Our results suggest strongly positive correlation between the SCH and BSS categories, whereas either group is negatively correlated with the CCX category; see Fig. 3b. We leveraged such insights in employing our forecasting method.

### Forecasting performance comparisons

We trained the prediction models of our proposed Bayesian forecasting method on the two time series between January – August 2017 for the three selected NHs. To evaluate the performance of our method, we conducted a comprehensive comparative study based on the forecasting results over a three-month forecast horizon (i.e., September, October, November 2017) and with the length of the rolling forecast window being one month. We made a forecast at the beginning of each month with the use of additional data made available up to that time within the previous rolling window. For each NH’s dataset, two quantities of service needs (i.e., resident volume of each acuity category and staff time of each caregiver type) are predicted over the entire forecast horizon.

We computed the median over 1000 samples of the daily prediction with our forecasting method. It is worth noting that while our method essentially relies on moment matching, i.e., updating the mean and variance of both the latent state vector and the model parameter vector, we can characterize the predictive distribution through estimating all relevant distribution parameters. With the full characterization repeatedly, we can sample a large number of predicted values and take their sample median for comparison.

**Fig. 4:**
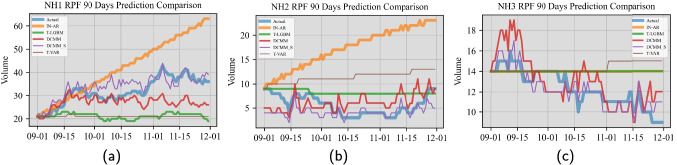
Comparison of 90-day point forecasts on daily RPF resident volume. The horizontal axis shows dates (Sep 2017 to Nov 2017) on which we performed the comparative testing. Each subfigure corresponds to one test NH

**Fig. 5:**
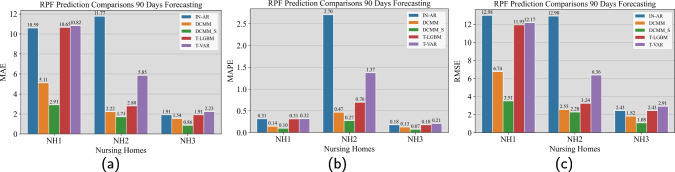
Comparison of prediction models (DCMM, DCMM_S, IN-AR, T-LGBM, and T-VAR) on forecasting NH1’s RPF resident volume 90 days prior. The performance (forecast errors) is measured by MAE, MAPE, and RMSE, and reported in Subfigures (a, b, c), respectively

We subsequently compared different prediction methods and compared the predicted values against the actual records. For each predicted value, we computed the forecast error ($e_k$), which is the difference between the observation value ($x_k$) and the predicted value ($\hat{x}_k$) for the *k*-th day in the forecasting window. We subsequently used mean absolute error (MAE), mean absolute percentage error (MAPE), and root mean square error (RMSE) as performance evaluation metrics to assess the accuracy of each considered prediction model. These metrics are presented as:

$$\begin{aligned} \text {MAE}= & \frac{1}{K}\sum _{k=1}^K \vert e_k\vert ,\\ \text {MAPE}= & \frac{1}{K}\sum _{k=1}^K \frac{\vert e_k\vert }{x_k},\\ \text {RMSE}= & \sqrt{\frac{\sum _{k=1}^K (e_k)^2 }{K}}. \end{aligned}$$

#### Resident volume forecasting

Our proposed Bayesian forecasting method has two prediction models which differ by whether shared information among groups is incorporated. The two version for the quantity of resident volume are termed as DCMM and DCMM_S, respectively, and they are detailed in Sections 4.1 and 4.3. In the actual implementation of our proposed forecasting method, we specify $F^{\text {D}}_{g,t}=1$, $\tilde{F}^{\text {D}}_{g,t}=1$ and $\boldsymbol{G}^{\text {D}}_{g,t}=\boldsymbol{I}$ in Eqs. 3 and 4, respectively, as well as $\boldsymbol{\omega }^{\text {D}}_{g,t}$ as a Gaussian noise, in the resident volume modeling. In addition, we considered several state-of-the-art prediction models as benchmarks for the forecasting comparison. They are integer-valued autoregressive model combined with Poisson distribution [53] (labeled as IN-AR), truncated vector autoregressive model [7] (labeled as T-VAR), and truncated light gradient-boosting machine model [28] (labeled as T-LGBM). For information on training the benchmark models, please see Appendix B.1. For the interest of space, we only present the point forecast comparisons for the resident volume of the RFP category in the main text; see Figs. 4 and 5. We present the comparison results on the same quantity for the CCX category and over all 6 categories in Appendix.

From Fig. 4a displaying the comparison results for NH1, we made the following observations. The IN-AR model under the stationary assumption yielded linear trending estimates on the forecasting quantity, but failed to capture the non-stationary oscillation. Meanwhile, the T-VAR and T-LGBM models failed to capture the growing trend. Both the DCMM and DCMM_S models effectively capture the weekly/biweekly oscillation pattern. Between the two DCMM_S could follow the slightly growing trend better. Figure 4b displays the comparison results for NH2. The observed data exhibit fluctuating patterns over time, with a slight initial decline followed by a subsequent slight increase. The results suggest that DCMM and DCMM_S could well capture both patterns. We also made similar observations on the benchmark models. Figure 4c displays the comparison results for NH3. The observed data exhibit regular oscillations superimposed on a declining trend. The results again suggest that DCMM and DCMM_S could well capture both patterns. We also made similar observations on the benchmark models. Note that the forecasts of IN-AR (orange line) and T-LGBM (green line) are identical over the entire forecast horizon.

The comparison results displayed in Fig. 5 further confirmed that the DCMM_S model provides the lowest MAE, MAPE, and RMSE scores as opposed to the one without incorporation of shared information as well as benchmark models, for all three test NHs. The DCMM model was ranked second in all the cases. The relative performance improvement against the benchmarks may be dependent upon the NH type. Similar observations were made from most comparisons related to predicting the resident volumes of the CXX category and the entire facility; see Figs. 9 – 14. The only exception appeared in the forecasting of NH2 where DCMM_S only trailed the IN-AR model.

**Fig. 6:**
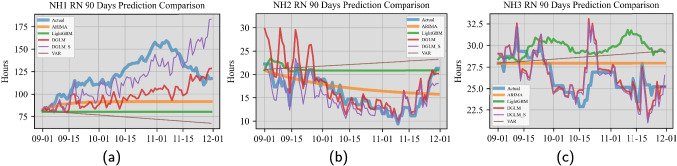
Comparison of 90-day point forecasts on daily RN staff hours. The horizontal axis shows dates (Sep 2017 to Nov 2017) on which we performed the comparative testing. Each subfigure corresponds to one test NH

**Fig. 7:**
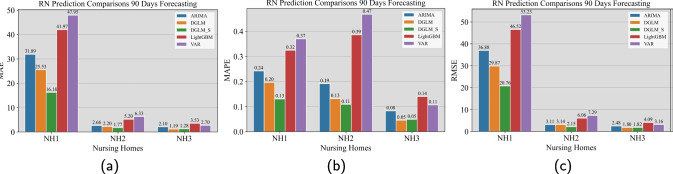
Comparison of prediction models (DGLM, DGLM_S, ARIMA, LGBM, and VAR) on NH1’s RN staff time (in hours) 90 days prior. The performance (forecast errors) is measured by MAE, MAPE, and RMSE, and reported in Subfigures (a, b, c), respectively

#### Staff time forecasting

Similar to the previous section, we considered two models for our proposed forecasting method. The two models for the quantity of staff time are termed as DGLM and DGLM_S, respectively, and they are detailed in Sections 4.2 and 4.3. In the actual implementation of our proposed forecasting method, we specified $\tilde{F}_{r,t}^{\text {C}}=1$, $F_{r,t}^{\text {C}}=1$, $\boldsymbol{G}^{\text {C}}_{r,t}=\boldsymbol{I}$, and $\boldsymbol{\omega }^{\text {C}}_{r,t}$ as a Gaussian noise, in the staff time modeling. The considered benchmark models include ARIMA, VAR, and LightGBM. For information on training the benchmark models, please see Appendix B.2. For the interest of space, we only present the point forecasts comparison for RN staff time in the three NHs in the main text; see Fig. 6 and 7. We present the comparison results on the same quantity for LPN and CNA in Appendix.

**Fig. 15:**
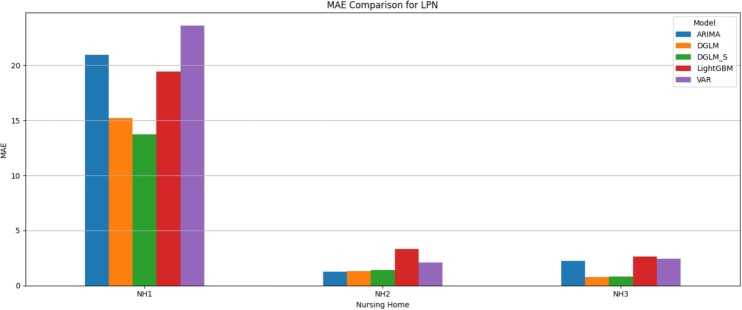
Comparison of the prediction models (i.e., DGLM, DGLM_S, ARIMA, LightGBM, and VAR) on the staff time (in hours) of LPN. The 90-day forecast errors are measured by MAE. The three segments are for the three test NHs, respectively

**Fig. 16:**
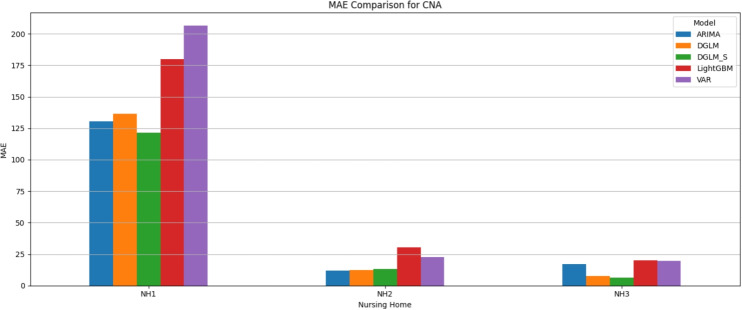
Comparison of the prediction models (i.e., DGLM, DGLM_S, ARIMA, LightGBM, and VAR) on the staff time (in hours) of CNA. The 90-day forecast errors are measured by MAE. The three segments are for the three test NHs, respectively

**Fig. 17:**
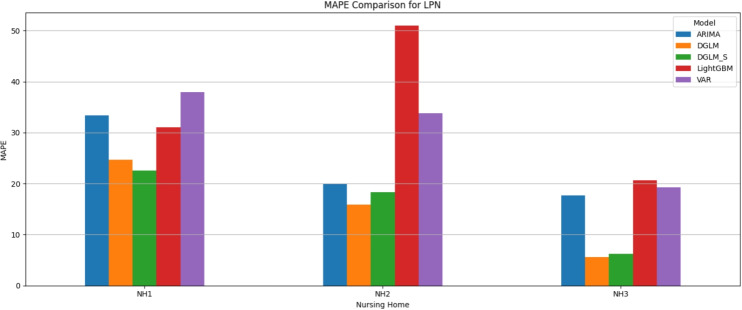
Comparison of the prediction models (i.e., ARIMA, DGLM, DGLM_S, ARIMA, LightGBM, and VAR) on the staff time (in hours) of LPN. The 90-day forecast errors are measured by MAPE. The three segments are for the three test NHs, respectively

**Fig. 18:**
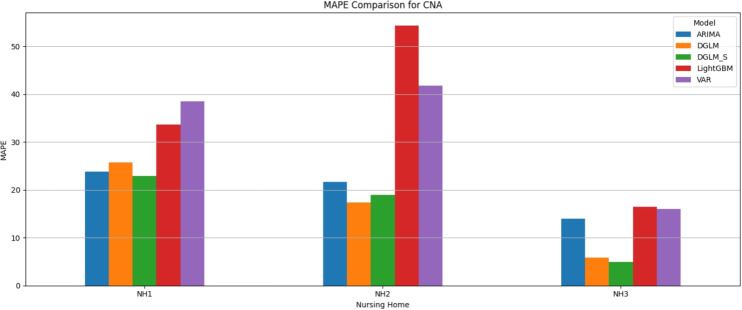
Comparison of the prediction models (i.e., DGLM, DGLM_S, ARIMA, LightGBM, and VAR) on the staff time (in hours) of CNA. The 90-day forecast errors are measured by MAPE. The three segments are for the three test NHs, respectively

**Fig. 19:**
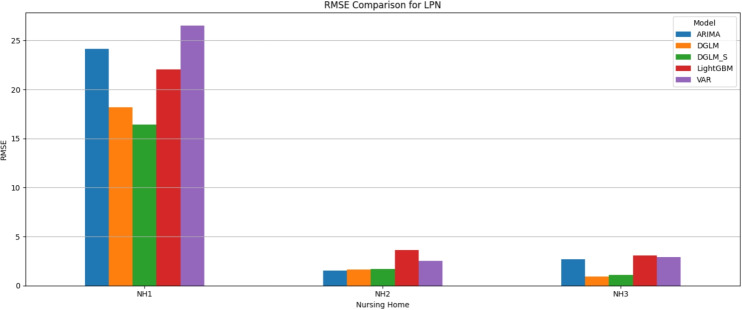
Comparison of the prediction models (i.e., DGLM, DGLM_S, ARIMA, LightGBM, and VAR) on the staff time (in hours) of LPN. The 90-day forecast errors are measured by RMSE. The three segments are for the three test NHs, respectively

**Fig. 20:**
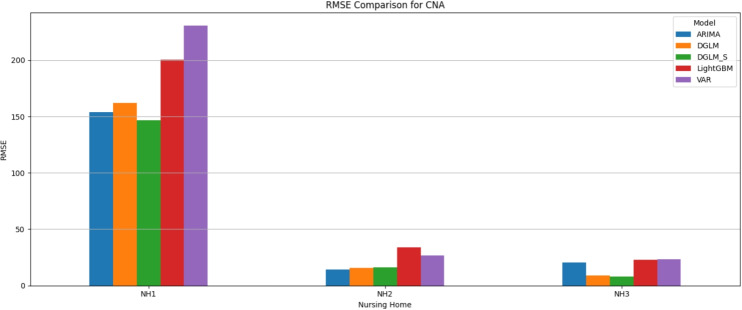
Comparison of the prediction models (i.e., DGLM, DGLM_S, ARIMA, LightGBM, and VAR) on the staff time (in hours) of CNA. The 90-day forecast errors are measured by RMSE. The three segments are for the three test NHs, respectively

From Fig. 6 displaying the point forecast comparison results, we made the following observations. For NH1, the actual RN staff time data present regular weekly/biweekly oscillations and a pattern of first growing and then slightly deceasing. The results suggested that both the DGLM and DGLM_S models effectively captured the oscillating pattern. Both models also captured the growing trend but could not capture the slightly decreasing trend. Between the two, DGLM_S performed slightly better. Nevertheless, the benchmark models failed to capture either pattern. For NH2, the actual RN staff data has the regular oscillations and a pattern of first decreasing and then slightly increasing. The results suggested that both the DGLM and DGLM_S models effectively captured both patterns. We drew the same conclusions for NH3. Moreover, Fig. 7 show consistent comparison results among the three metrics. DGLM and DGLM_S outperform the benchmark models for all three NHs. Similar observations were made from the comparisons related to the LPN and CNA staff time forecasting; see Figs. 15 – 20.

In conclusion, our results suggest that (1) both DGLM and DGLM_S are reasonably good compared to many conventional methods; and (2) the ARIMA model can be good when trending is not noticeable in the data.

## Conclusion

In this paper, we present a novel Bayesian estimation approach for NH service need forecasting, which can effectively address non-stationary evolving patterns at both discrete and continuous scales in resident volume and staff time, respectively. In addition, our proposed forecasting method is able to handle excess zeros in the discrete case and allows information sharing from correlated service need data. Further, we embed the idea of Bayesian estimation and sequential updating in a multi-step forecasting framework with rolling window along a forecasting horizon. This ensures convenient model updating and timely capture of non-stationary evolving patterns. We test our approach on a few NHs, suggesting the superiority of our method.

Our study highlights the limitations of benchmark models in handling non-stationary in the data. With the capability of incorporating shared information when modeling the multi-dimensional non-stationary time-series, the DCMM_S and DGLM_S models can help address these challenges, resulting in consistent performance improvement or at least comparable performance. Consequently, we believe that our Bayesian estimation approach is a significant contribution to the field of NH manpower and staffing research, providing an in-depth understanding of service need assessment and resource utilization, leading to better decision-making and improved quality of life for NH residents.

The current study has several limitations. First, MDS data used to train the model may be considered low-quality. The resident assessment data recorded at NHs may be incomplete, biased, and contain errors, which can lead to inaccurate predictions. In addition, the current service need data we obtained are realized admissions, which depend upon the facility’s admissions policies, the discharge policies of upstream facilities (hospitals), and perhaps residents’ choice preferences as well. In the future, we will extend the current work to incorporate these dimensions as endogenous variables in the modeling or acquire additional data on these dimensions to improve the modeling.

From the viewpoint of forecasting methodology, our proposed approach may not always be superior to others for different populations or NH types. This can largely be due to insufficient and inefficient tuning of the proposed prediction models. To improve the parameter tuning efficiency, we will focus on using efficient hyperparameter optimization techniques, leveraging surrogate models, and optimizing resource allocation. Specifically, in future research, we will consider Bayesian optimization, random search with early stopping, and distributed computing strategies. We will also develop protocols for leveraging NH domain expertise to monitor model training and provide prompt feedback for parameter tuning.

Finally, our forecasting only relies on the MDS data to specify residents’ acuity retrospectively. While we identified a few NH characteristics (e.g., number of beds) as key forecasting features earlier on and selected the test NHs accordingly, we did not incorporate such characteristics and potentially other static features in the forecasting. Such incorporation is expected to improve the performance further, whose utility will be explored in future research.. In addition, without real-time assessment data could limit the usefulness of the acuity calculations for accurate service operations management. In our future work, we will incorporate additional sources of real-time or near real-time data (e.g., Real-time Quality Measure and Certification and Survey Provider Enhanced Report [33]). Further, additional data may include data collected from wearable devices, which improves the timeliness of the assessment data but incurs computational challenges in analyzing the data of higher resolution and increased dimensionality.

## Figures and Tables

**Fig. 8:**
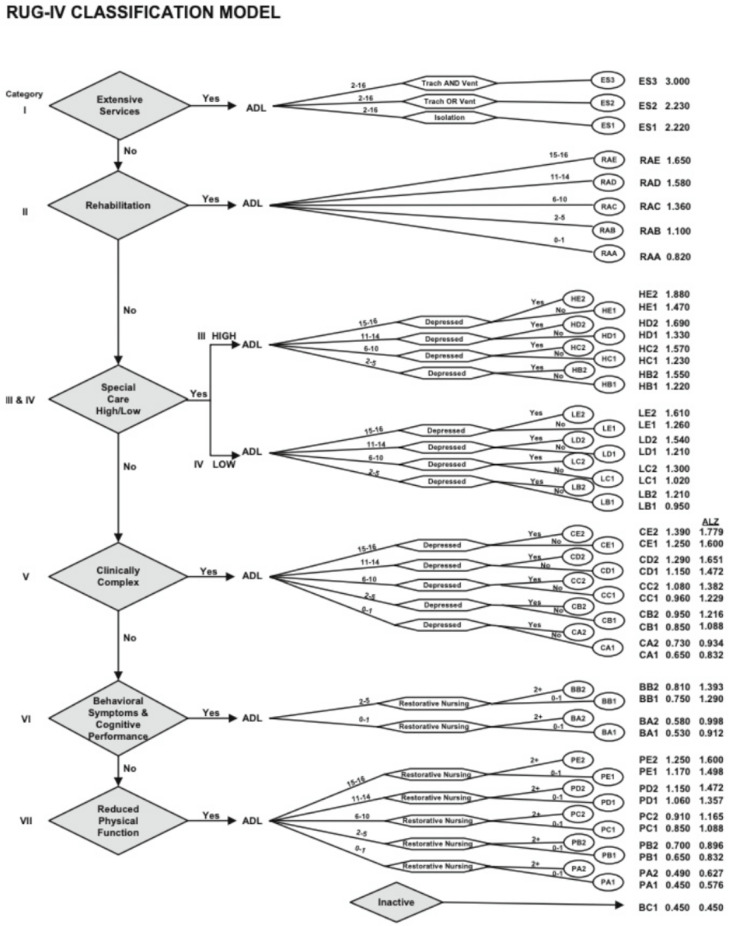
RUG-IV classification model. Each resident is assigned to one of the 48 RUGs

**Fig. 9:**
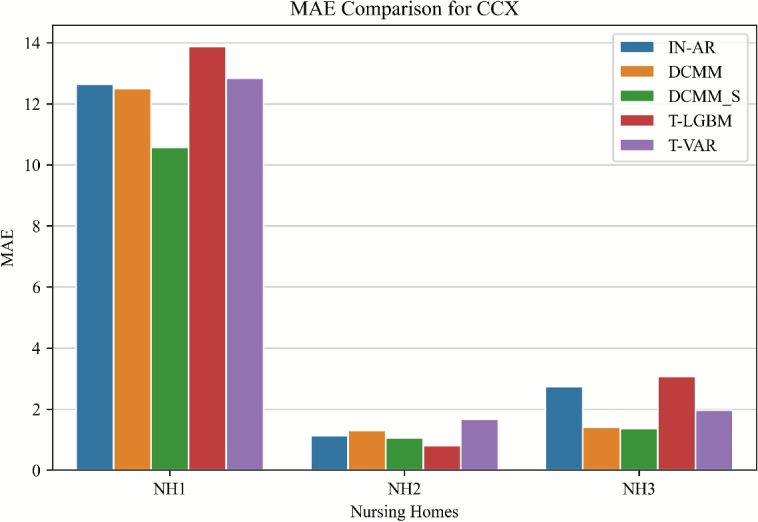
Comparison of the prediction models (i.e., DCMM, DCMM_S, IN-AR, T-LGBM, and T-VAR) on the resident volume of the CCX category. The 90-day forecast errors are measured by MAE. The three segments are for the three test NHs, respectively

**Fig. 10:**
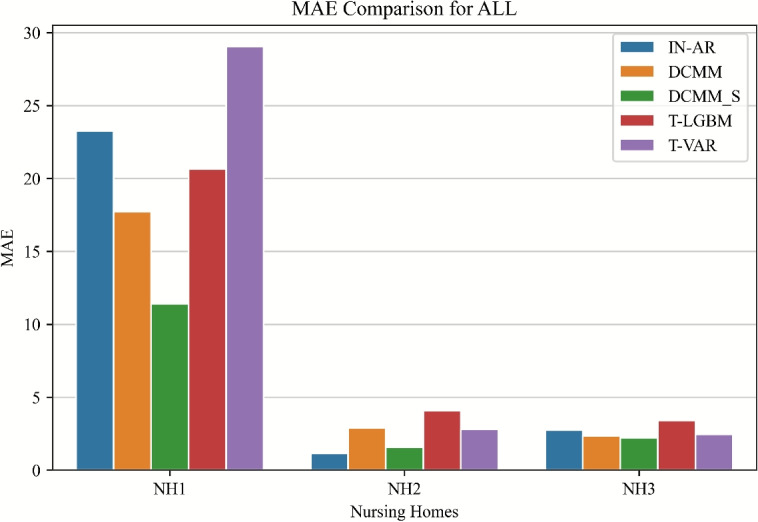
Comparison of the prediction models (i.e., DCMM, DCMM_S, IN-AR, T-LGBM and T-VAR) on the resident volume over all acuity categories. The 90-day forecast errors are measured by MAE. The three segments are for the three test NHs, respectively

**Fig. 11:**
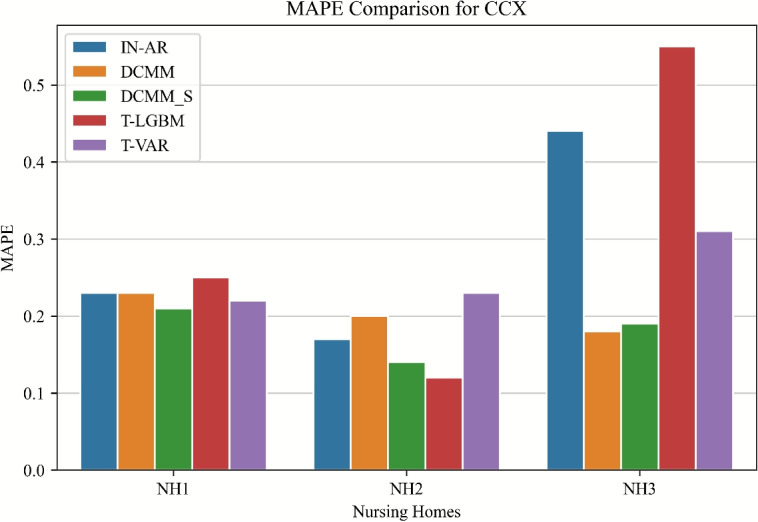
Comparison of the prediction models (i.e., DCMM, DCMM_S, IN-AR, T-LGBM, and T-VAR) on the resident volume of the CCX category. The 90-day forecast errors are measured by MAPE. The three segments are for the three test NHs, respectively

**Fig. 12:**
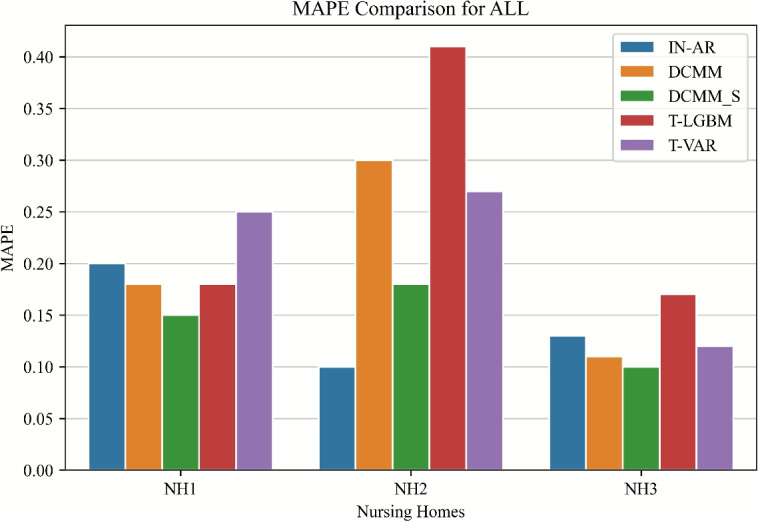
Comparison of the prediction models (i.e., DCMM, DCMM_S, IN-AR, T-LGBM and T-VAR) on the resident volume over all acuity categories. The 90-day forecast errors are measured by MAPE. The three segments are for the three test NHs, respectively

**Fig. 13:**
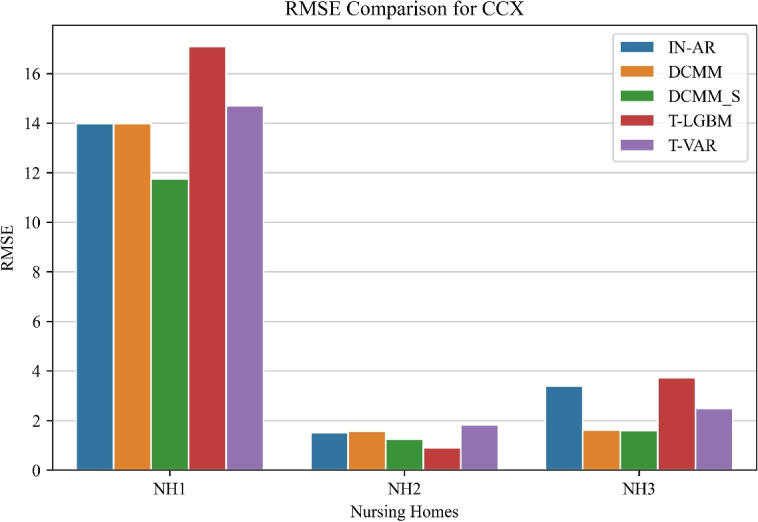
Comparison of the prediction models (i.e., DCMM, DCMM_S, IN-AR, T-LGBM, and T-VAR) on the resident volume of the CCX category. The 90-day forecast errors are measured by RMSE. The three segments are for the three test NHs, respectively

**Fig. 14:**
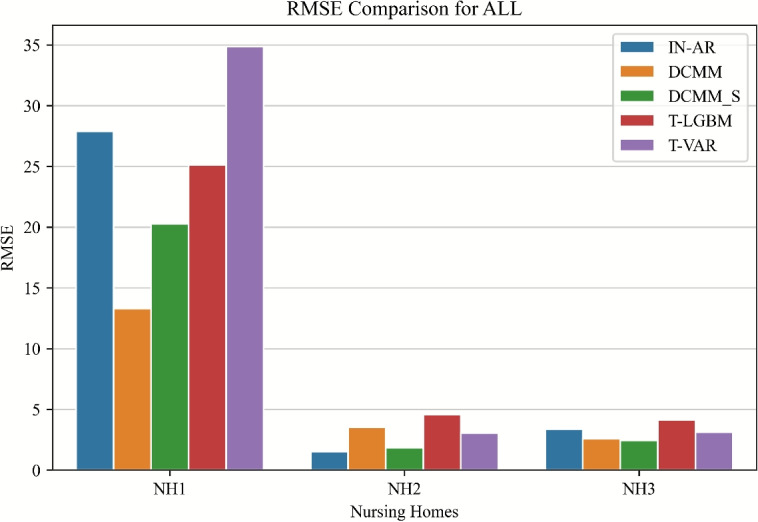
Comparison of the prediction models (i.e., DCMM, DCMM_S, IN-AR, T-LGBM, and T-VAR) on the resident volume over all acuity categories. The 90-day forecast errors are measured by RMSE. The three segments are for the three test NHs, respectively

## Appendix A: Service need classification and mapping

**Table 2:** CMS-recommended staff time (in hours) per resident day following RUG-IV guidelines and PDPM specifications. In our case studies, we only analyzed data for the six acuity categories (Category I, Categories III – VIII) where residents need skilled services from all three types of caregivers, which presents a higher level of data complexity. Residents in Category II, rehabilitation, need more specialized care not from nursing staff, thus being omitted in our study

Acuity categories	RUG	PDPM	RN	LPN	CNA
Extensive services (ESS)	ES3	ES3	2.39	1.69	3.6
	ES2	ES2	1.81	1.43	3.6
	ES1	ES1	1.35	0.96	3.6
Special care high (SCH)	HE2	HDE2	1.2	0.99	3.6
	HD2	HDE2	1.2	0.99	3.6
	HE1	HDEI	1.2	0.99	3.6
	HD1	HDEI	1.2	0.99	3.6
	HC2	HBC2	1.53	0.7	3.2
	HB2	HBC2	1.53	0.7	3.2
	HC1	HBC1	1.53	0.7	3.2
	HB1	HBC1	1.53	0.7	3.2
Special care low (SCL)	LE2	LDE2	1.2	0.99	3.6
	LD2	LDE2	1.2	0.99	3.6
	LE1	LDE1	1.2	0.99	3.6
	LD1	LDE1	1.2	0.99	3.6
	LC2	LBC2	1.53	0.7	3.2
	LB2	LBC2	1.53	0.7	3.2
	LC1	LBC1	1.53	0.7	3.2
	LB1	LBC1	1.53	0.7	3.2
Clinical complex (SCC)	CE2	CDE2	1.22	0.7	3.6
	CD2	CDE2	1.22	0.7	3.6
	CE1	CDE1	0.96	0.78	3.6
	CD1	CDE1	0.96	0.78	3.6
	CC2	CBC2	0.99	0.67	3.2
	CB2	CBC2	0.99	0.67	3.2
	CA2	CA2	1	0.63	2.8
	CC1	CBC1	1	0.63	3.2
	CB1	CBC1	1	0.63	3.2
	CA1	CA1	1	0.63	2.8
Behavioral symptoms (BSC)	BB2	BAB2	0.75	0.55	3
	BA2	BAB2	0.75	0.55	3
	BB1	BAB1	0.75	0.55	3
	BA1	BAB1	0.75	0.55	3
Reduced physical function (RPF)	PE2	PDE2	0.75	0.55	3.6
	PD2	PDE2	0.75	0.55	3.6
	PE1	PDE1	0.75	0.55	3.6
	PD1	PDE1	0.75	0.55	3.6
	PC2	PBC2	0.75	0.58	3.2
	PB2	PBC2	0.75	0.58	3.2
	PA2	PA2	0.75	0.55	2.8
	PC1	PBC1	0.75	0.55	3.2
	PB1	PBC1	0.75	0.55	3.2
	PA1	PA1	0.75	0.55	2.8

## Appendix B: Specifications on the parameters of two benchmark models

### B.1 Resident volume forecasting

In this section, we discuss our training of the integer autoregressive (IN-AR) model and truncated vector autoregressive (T-VAR) models. In general, across the three NHs, NH1 demonstrates predictable and structured inter-category dependencies, particularly between RPF and other categories, suggesting T-VAR a valuable complement to IN-AR. NH2 shows meaningful but weaker interactions, with ALL acting as a central hub of variability. In contrast, NH3 exhibits low temporal complexity and minimal cross-category influence, where univariate IN-AR models are sufficient and T-VAR offers limited additional benefit. For a summary of the model parameters and learning outcomes, please review Table 3.

- NH1
  - IN-AR
    *: The best AIC value (655.995) comes from using a deep model i.e., AR(4), when predicting the resident volume of the CCX category, which indicates well-captured periodic fluctuations.
    *: To predict the resident volume of the RFP category, IN-AR(4) comes up to be the best fit. To predict the same quantity over all acuity categories, IN-AR(2) comes up to the best fit.
    *: For the resident volume of all acuity categories, the model results in a much higher AIC/BIC, which is likely due to its aggregate nature or noise.
  - T-VAR
    *: The residual correlation shows significant coupling between several studied acuity categories (e.g., RPF to CCX and ALL)
    *: More specifically, there are significant residual correlations (CXX and ALL 0.47, RPF and ALL: 0.58), which indicates shared dynamics between the different acuity categories.
- NH2
  - IN-AR
    *: The best AIC value (244.43) comes from using a light model, i.e., AR(2), when predicting the resident volume of the CCX category, which indicates stable behavior with minor long-memory effects.
    *: For the resident volume of the RPF and All categories, more complex models perform well, i.e., IN-AR(4) for the former and IN-AR(3) for the latter. This points to richer dynamics in these two categories.
  - T-VAR
    *: The residual correlation shows moderate coupling, i.e., stronger than NH3 but weaker than NH1. Significant terms are ALL to RPF (lag 2, 5, 7) and CCX to ALL (lag 1).
    *: More specifically, there are moderate residual correlations (ALL and CCX: 0.54, ALL and RPF: 0.58).
- NH3
  - IN-AR
    *: The best AIC value (-22.27) is negative when predicting the resident volume of the CCX category, which indicates excellent fit with low noise.
    *: This model is a good fit when predicting the resident volume for the RPF category and all the acuity categories. This suggests low variance, low autocorrelation series.
    *: To predict the resident volume over all acuity categories, the order of IN-AR is at the same level for the other NHs. To the contrary, for the RFP category, the order of the IN-AR model is lower as opposed to the other NHs.
  - T-VAR
    *: There is weak interdependency overall (e.g., ALL to CCX, RPF to ALL). But the effects are small.
    *: The residual correlation shows weak coupling than NH1 and NH2.

**Table 3:** IN-AR and T-VAR parameter values and training performances

NH	Model	Series	Order	AIC	BIC
		RFP	4	732.725	774.24
NH1	IN-AR	CCX	4	655.995	694.098
		ALL	2	1022.944	1047.191
NH1	T-VAR		lag = 7	0.573641	1.54527
		RFP	4	358.643	393.282
NH2	IN-AR	CCX	2	244.428	261.747
		ALL	3	619.895	661.461
NH2	T-VAR		lag = 7	−4.64976	−3.67813
		RFP	1	303.102	323.885
NH3	IN-AR	CCX	2	−22.273	12.365
		ALL	3	445.4	486.966
NH3	T-VAR		lag = 7	−6.61633	−5.6447

### B.2 Staff time forecasting

In this section, we discuss our training of the ARIMA and VAR models. In general, NH1 benefits the most from the multivariate modeling (i.e., VAR), whereas NH3 is well-described by simple univariate models (i.e., ARIMA). In all NHs, residuals are highly correlated, possibly due to shared institutional constraints or external events not captured in the models. For predictive purposes, LPN appears to be the most informative and stable caregiver type across the test NHs. For a summary of the model parameters and learning outcomes, please review Table 4.

- NH1
  - ARIMA
    *: Different caregiver types (RN, LPN, CNA) require different levels of autoregressive and seasonal components, suggesting heterogeneous temporal patterns.
    *: The CNA series has the highest AIC/BIC, suggesting its data are the hardest to model and/or the most volatile.
  - VAR
    *: There is significant interdependency. For example, the LPN series strongly influences the other two series (e.g., LPN to CNA with *p* = 0.012). This may reflect real-world staffing dynamics, perhaps because changes in LPN scheduling impact others.
    *: There are strong residual correlations (all > 0.95), which further confirms coordinated patterns across the staff types.
- NH2
  - ARIMA
    *: All series follow a similar AR(3) structure, possibly indicating a shared operational rhythm (e.g., shift cycles).
    *: The LPN series has the best model fit (lowest AIC/BIC), suggesting it is more predictable or consistently scheduled.
  - VAR
    *: There are weak coefficients across the board with no significant cross-type influence.
    *: There are strong residual correlations (all > 0.94), which likely indicates common external drivers (e.g., policies or calendar events), but no direct interactions.
- NH3
  - ARIMA
    *: All three series were best modeled by a random walk (0,1,0), or with minimal seasonal AR.
    *: There is very low model complexity (i.e., no AR or MA team leads to low AIC/BIC), indicating stationary or very smooth patterns.
  - VAR
    *: There are no significant coefficients at lag-1. Inter-type influence is statistically absent.
    *: The residual correlations remain high, but the data suggests staff types evolve independently with little fluctuation.

**Table 4:** ARIMA and VAR parameter values and training performances

NH	Model	Series	Order	AIC	BIC
		RN	(1,1,1)	1187.297	1204.742
NH1	ARIMA	LPN	(3,1,1)	835.209	866.609
		CNA	(1,1,1)	1864.963	1882.408
NH1	VAR		lag = 1	0.526565	0.700082
		RN	(3,0,0)	710.245	727.71
NH2	ARIMA	LPN	(3,0,0)	396.437	420.888
		CNA	(3,0,0)	1429.85	1454.31
NH2	VAR		lag = 1	−4.76219	−4.58868
		RN	(0,1,0)	510.77	514.259
NH3	ARIMA	LPN	(0,1,0)	215.543	219.032
		CNA	(0,1,0)	1249.773	1256.751
NH3	VAR		lag = 1	−6.05149	−5.87797

## Abbreviations

ADL: Activity of Daily Living
ARIMA: Autoregressive Integrated Moving Average
BSS: Behavior Symptoms
CCX: Clinical Complex
CMS: Center for Medicare and Medicaid
CNA: Certified Nursing Assistant
DCMM: Discrete Census Mixture Model
DCMM_S: Discrete Census Mixture Model with Information Sharing
DGLM: Dynamic Generalized Linear Model
DGLM_S: Dynamic Generalized Linear Model with Information Sharing
ESS: Extensive Services
IN-AR: Integer-valued Autoregressive
LGBM: Light Gradient Boosting Machine
LPN: Licensed Practical Nurse
MAE: Mean Absolute Error
MAPE: Mean Absolute Percentage Error
MDS: Minimum Data Set
NH: Nursing Home
NPI: National Provider Identifier
OM: Operations Management
PDPM: Patient-driven Payment Model
RPF: Reduced Physical Function
RMSE: Root Mean Square Error
RN: Registered Nurse
RUG-IV: Resource Utilization Group Version IV
SCH: Special Care High
SCL: Special Care Low
T-LGBM: Truncated Light Gradient Boosting Machine
T-VAR: Truncated Vector Autoregressive
VAR: Vector Autoregressive
